# Associations of microbiota and toll-like receptor signaling pathway in esophageal adenocarcinoma

**DOI:** 10.1186/s12885-016-2093-8

**Published:** 2016-02-02

**Authors:** Ali H. Zaidi, Lori A. Kelly, Rachael E. Kreft, Mark Barlek, Ashten N. Omstead, Daisuke Matsui, Natalie H. Boyd, Kathryn E. Gazarik, Megan I. Heit, Laura Nistico, Pashtoon M. Kasi, Tracy L. Spirk, Barbara Byers, Emily J. Lloyd, Rodney J. Landreneau, Blair A. Jobe

**Affiliations:** Esophageal and Lung Institute, Allegheny Health Network, 4600 North Tower, 4800 Friendship Avenue, Pittsburgh, PA 15224 USA; Center of Excellence in Biofilm Research, Allegheny Health Network, Pittsburgh, PA USA; Department of Medicine, Mayo Clinic, Rochester, Minnesota USA

**Keywords:** Esophageal adenocarcinoma, Microbiota, Toll-Like Receptors, Levrat model

## Abstract

**Background:**

Toll-like receptors (TLRs) recognize known molecules from microbes and have an established role in tumorigenesis. Using a rat model of esophageal adenocarcinoma, and human clinical samples, we investigated genes central to TLR-mediated signal transduction and characterized the esophageal microbiome across the spectrum of esophageal adenocarcinoma carcinogenesis.

**Methods:**

We surgically induced bile/acid reflux in rats and their esophagi were harvested at 40 weeks post-surgery. Tissue samples from the model were selected for gene expression profiling. Additionally, for rat and human samples microbiome analysis was performed using PCR-ESI-MS-TOF technology with validation by fluorescence in situ hybridization.

**Results:**

Gene expression results in the rat model indicated a significant upregulation of TLRs 1-3, 6, 7 and 9 in EAC compared to normal epithelium. PCR-ESI-MS-TOF analysis revealed a prevalence of Escherichia coli in Barrett’s esophagus (60 %) and esophageal adenocarcinoma (100 %), which was validated by fluorescence in situ hybridization. In the human clinical samples, *Streptococcus pneumonia* was detected in high abundance in gastroesophageal reflux disease and Barrett’s esophagus (50–70 %) in comparison to tumor adjacent normal epithelium, dysplasia, and esophageal adenocarcinoma (20–30 %). *E. coli* was detected in the Barrett’s esophagus and esophageal adenocarcinoma groups but was absent in the tumor adjacent normal epithelium, dysplasia, and the gastroesophageal reflux disease groups.

**Conclusions:**

We demonstrated an association between the TLR signaling pathway and *E. coli* hinting towards possible early molecular changes being mediated by microbes in the rat model of esophageal adenocarcinoma carcinogenesis. Studies on human clinical samples also corroborate results to some extent; however, a study with larger sample size is needed to further explore this association.

**Electronic supplementary material:**

The online version of this article (doi:10.1186/s12885-016-2093-8) contains supplementary material, which is available to authorized users.

## Background

The incidence of esophageal adenocarcinoma (EAC) has been on the rise over the past several decades [1]. Despite some advances in the understanding of its pathogenesis and treatment modalities, the 5 year survival for locally advanced EAC is still dismal (less than 25 %) [2].

Barrett’s esophagus (BE) is the established precursor to EAC that progresses through a metaplasia-dysplasia-carcinoma sequence. BE is characterized by replacement of normal stratified squamous epithelium by more intestinal-like, simple columnar epithelium containing goblet cells. It is postulated that conversion to a more intestinal-like epithelium is an attempt to provide protection from acid and bile induced tissue damage in the esophagus. Intestinal epithelial cells (IECs) provide this protection in the gut by allowing colonization of the intestinal tract by commensal bacteria which aid in digestion. This markedly influences the development and function of the mucosal immune system [3].

Recently, several studies demonstrate the importance of specifically the innate microbial recognition by immune and nonimmune cells in the gut. Paradoxically, either diminished or exacerbated innate immune signaling may trigger the breakdown of intestinal homeostasis, leading possibly to disease [4]. This link between bacterial signaling and innate immune host response has now been demonstrated for several gastrointestinal (GI) malignancies including esophageal cancer [5–11].

Given these findings, there has been an increased interest in the role of innate immunity in tumorigenesis and the need for identifying new potential targets [12]. Within this domain comes the role of toll-like receptors (TLRs). TLRs constitute a family of highly conserved, pattern-recognition receptors that recognize known molecules from microbes as pathogen-associated molecular patterns (PAMPs) or danger-associated molecular patterns (DAMPs) that are endogenous molecules released from necrotic or dying cells [13–16]. Different TLRs serve as receptors for diverse ligands including bacterial cell wall components, viral double-stranded RNA, small-molecule antiviral or immunomodulatory compounds [17], flagellin and bacterial DNA. Since TLRs connect innate and adaptive immune responses, they represent a significant and potentially linking element between inflammation and cancer [18].

The goal of our present study was to evaluate the potential links between microbial signaling, host response and EAC progression by characterizing the rat esophageal microbiome and focusing on profiling the expression of genes central to TLR-mediated signal transduction in a rat reflux model. Additionally, we characterized the microbiota in human EAC progression.

## Methods

### Ethics statement

The Institutional Animal Care and Use Committee (IACUC) at Allegheny Health Network approved the respective animal study protocols, all animals used in this study were cared for, and all procedures were in compliance with the “Guide for the Care and Use of Laboratory Animals”. All animals were euthanized by carbon dioxide inhalation.

Additionally, human samples were obtained under written consent and the study was conducted in accordance with the Declaration of Helsinki. The study was approved by the Internal Review Board (IRB) at Allegheny Health Network under IRB Protocol 14-048 and the assigned IRB Protocol number for written consent was 12-036.

### Experimental design

**Fig. 1:**
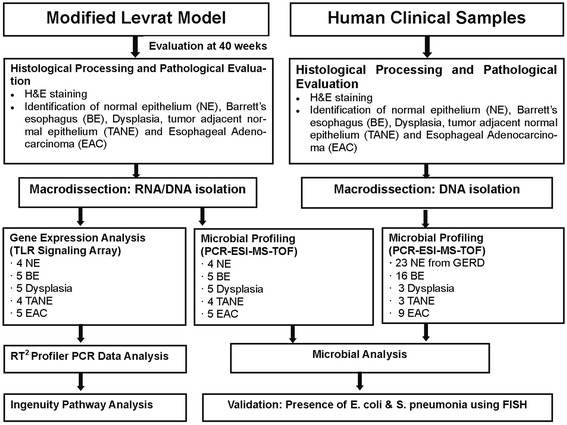
Study Schema outlining the major steps in the experimental design

Study schema outlining the major steps in the experimental design is represented in Fig. 1.

### Levrat model

The Levrat model was used to create a surgical end-to-side esophagojejunal anastomosis in 6–8 week-old, 300 g male Sprague-Dawley rats (Harlan Laboratories, Indianapolis, IN) as previously described [19]. The animals were closely monitored post-operatively and weighed weekly. Control animals (no surgery) were included in the study. All animals were euthanized at 40 weeks post-operatively by carbon dioxide inhalation. If rats experienced a 25 % or greater weight loss after immediate postoperative period or fit alternate euthanasia criteria, they were sacrificed prior to 40 weeks.

### Histological processing and pathological evaluation

Upon necropsy, the entire esophagus and jejunum, to a length approximately 1 cm distal to anastomosis, were collected and opened longitudinally. Collected tissue specimens were snap frozen in Tissue-Tek® O.C.T. compound (Sakura® Finetek, Torrance, CA; #4583), sectioned into 5 μm slides, and stained with hematoxylin and eosin (H&E). The H&E stained slides were reviewed by two pathologists to identify areas of normal epithelium (NE), Barrett’s esophagus (BE), dysplasia, tumor adjacent normal epithelium (TANE) and esophageal adenocarcinoma (EAC). Discrepancies in interpretation were resolved by consensus conference between the 2 pathologists. EAC was characterized by mucinous, dysplastic glandular cell growth with atypical nuclei and invasion through the basement membrane. Dysplasia was characterized by elongated nuclei; invasive to submucosa and/or muscle. BE was characterized by the presence of goblet cells in the mucosa. TANE was characterized by regular mucosa, submucosa and muscle layers. NE was collected from non-surgical rats.

### Molecular analyses

#### Macrodissection

NE, TANE, BE, dysplasia and EAC tumor were macrodissected from snap frozen specimens harvested from the Levrat models and patients for molecular analyses. Briefly, two tubes of five 40 μm sections were cut in a cryostat for mRNA and biofilm analysis, respectively. The areas of interest were dissected using a cold, RNase-free razor and placed in QIAzol buffer (Qiagen, Valencia, CA; #79306) for RNA extraction or ATL buffer (Qiagen, Valencia, CA; #19076) for DNA isolation. The same number of sections were cut from NE specimens and placed directly into QIAzol or ATL buffer.

#### MRNA profiling

RNA was isolated using the miRNeasy Kit (Qiagen, Valencia, CA; #217004). RNA concentration was spectrophotometrically assessed on the SpectraMax M2e plate reader (Molecular Devices, Sunnyvale, CA) and RNA quality was assessed by Bioanalyzer (Agilent, Santa Clara, CA) RIN (RNA Integrity Number) . Four samples each of NE and TANE, and 5 samples each of BE, dysplasia and EAC tumor were selected for mRNA profiling using the RT^2^ Profiler Rat Toll-like Receptor Signaling Pathway Array (SA Bioscience, Frederick, MD; #330231 PARN018Z). Briefly, 500 ng of total RNA was treated for gDNA contamination then reverse transcribed in a total volume of 10ul at 42 °C for 15 min. This was followed by inactivation of reverse transcriptase at 95 °C for 5 min using the RT^2^ First Strand Kit (Qiagen, Valencia, CA; #330401) according to manufacturer recommendations. PCR was performed on cDNA using the PARN018Z Array and RT^2^ SYBR Green ROX qPCR Master Mix (Qiagen, Valencia, CA; #330520). Cycling parameters for PCR were: 95 °C for 10 min followed by 40 cycles of 95 °C for 15 s, 60 °C for 1 min, 95 °C for 15 s. Dissociation curve analysis cycling parameters were: 95 °C for 15 s, 60 °C for 1 min, 95 °C for 15 s. Data was normalized to Ribosomal protein, large, P1 (Rplp1) and expression calculated by the delta-delta-Ct (2^-ΔΔCT^) method [20]. The top differentially expressed mRNAs were identified using RT^2^ Profiler PCR Array Data Analysis v.3.5.

#### DNA extraction and microbial detection

For DNA extraction, the tissue was placed into a microcentrifuge tube (Axygen Scientific,Union City, CA; # MCT-175-L-C) containing 270 uL of ATL Lysis buffer (Qiagen, Valencia, CA; #19076) and 30uL proteinase K (Qiagen, Valencia, CA; #19131). Samples were incubated at 56 °C until lysis of the tissue was achieved, approximately 12–36 h. Next 100 μl of a mixture containing 50 μl each of 0.1 mm and 0.7 mm Zirconia beads (Biospec, Bartlesville, OK; # 11079101z, 11079107zx respectively) was added to the samples which were then homogenized for 10 min at 25 Hz using a Qiagen TissueLyser (Qiagen, Valencia, CA; #85300) . DNA from the lysed sample was then extracted using the Qiagen DNeasy Tissue kit (Qiagen, Valencia, CA; #69506).

For microbial detection, 10 μl of the isolated DNA sample was loaded per well onto the BAC detection PCR plate (Abbott Molecular, Des Plains, IL; # PN 05 N13-01). The BAC detection plate is a 96 well plate which contains 16 primers that survey all bacterial organisms by using the omnipresent loci (e.g. 16S rRNA gene sequence); while some are targeted to specific pathogens of interest (e.g. the Staphylococcus-specific *tuf*B gene). The plate also includes primers for the detection of *Candida* species and some antibiotic resistance markers (e.g. mecA, vanA, vanB, and KPC). An internal calibrator of synthetic DNA template is also included in each assay, controlling for false negatives (e.g. from PCR inhibitors) and enabling a semi-quantitative analysis of the amount of template DNA present. PCR amplification was carried out and the products were desalted in a 96-well plate and sequentially electro-sprayed into a mass spectrometer. The spectral signals were processed to determine the identities of the pathogens and their relative concentrations within the processed tissue [21].

#### Fluorescent in situ hybridization (FISH)

Aliquots of the tissue specimens were fixed with fresh 4 % paraformaldehyde and incubated for 2–4 h at 4 °C. After the incubation, the specimen was centrifuged and the supernatant removed. This process was repeated twice with Hank’s Salt Saline Solution (HBSS). Next, the samples were resuspended in 50 % ethanol-PBS solution and stored at −20 °C for evaluation with FISH. FISH was performed as described by Nistico et al. (41) using species-specific fluorescent 16 s rRNA probes. *Escherichia coli (E. coli)* was targeted using probe sequence “GCA TAA GCG TCG CTG CCG” [22] and *Streptococcus pneumonia (S. pneumonia)* was targeted using “GTG ATG CAA GTG CAC CTT” [23].

Samples were observed with Confocal Scanning Laser Microscopy (CSLM) imaging using a Leica DM RXE microscope attached to a TCS SP2 AOBS confocal system (Leica Microsystems, Exton, PA) using a 63X (NA1.2 ) water immersion lens.

### Processing of human clinical samples

A total of 32 esophageal and 22 gastric samples were collected from 28 patients and were snap frozen in O.C.T. The specimens were sectioned onto 5 μm slides, stained with hematoxylin and eosin (H&E), and reviewed by two pathologists, as described earlier, for both microbial detection and FISH. Areas of TANE (3), BE (13), dysplasia (3) and EAC (5) were identified in the esophageal disease groups while 8 areas of NE were identified in GERD specimens. Normal gastric epithelium was identified in 22 gastric specimens from patients independent of disease state.

### Sample size and statistical analysis

All studies were discovery pilot studies therefore sample size calculations were not applicable.

Statistical analyses were performed using SPSS software (IBM, Armonk, NY, Version 20). A p-value < 0.05 was considered statistically significant. Independent two-tailed T-tests with two-sample equal variance were used to compare mean gene expression profiles of TANE, BE, dysplasia and EAC to NE. For canonical pathway analysis IPA software was used [24].

## Results

### Modified Levrat rat model

A total of 37 animals were included in the present study by undergoing esophagojejunostomy as described above. Twenty-six rats survived to 40 weeks (29.8 % mortality). Eleven died (three-died of unknown cause, one-suffered postoperative anastomotic leak, three-died of respiratory complications, and four-died of esophageal disease). Of the 26 rats that survived to 40 weeks, 19 were chosen for macro dissection based on the presence of TANE (4), BE (5), dysplasia (5) and EAC (5). All areas were confirmed on pathology. Four normal control animals were also included in order to collect NE (4).

### MRNA analysis in the rat model

**Fig. 2:**
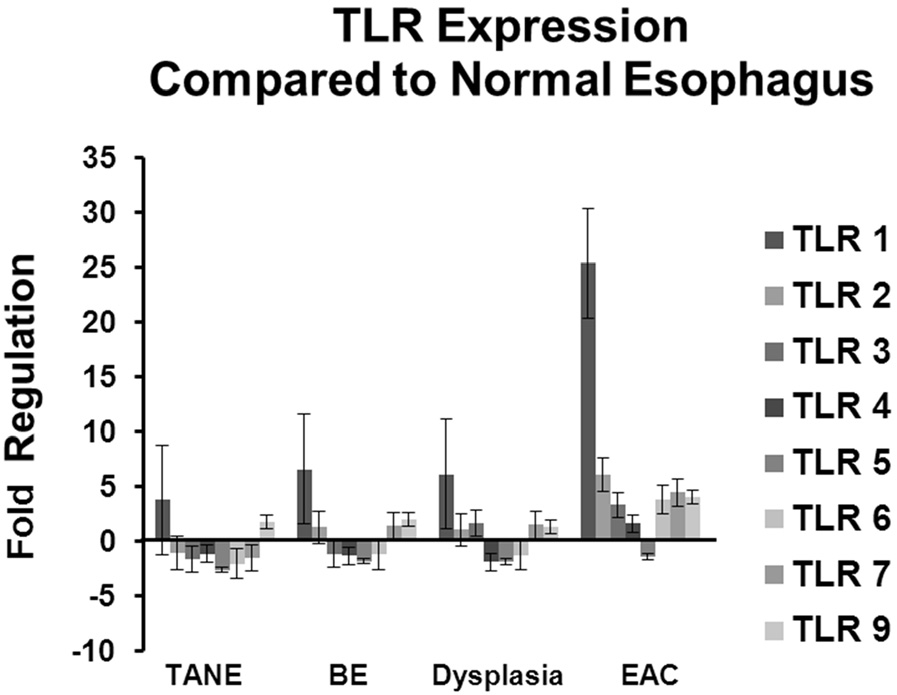
Toll-like receptor expression in TANE, BE, dysplasia and EAC compared to normal esophageal epithelium (NE). Significant upregulation of TLRs 1–3, 6, 7 and 9 are observed in EAC compared to normal epithelium

RT^2^ Profiler PCR Array Data Analysis v.3.5. identified TLRs 1-3, 6, 7 and 9 as significantly upregulated in EAC compared to NE in the rat model. TLR 1 was significantly upregulated in BE, dysplasia and TANE. TLR 5 and TLR 6 were significantly down regulated in TANE (Fig. 2). Thirty one genes involved in the TLR signaling pathway were significantly dysregulated in EAC, 27 in dysplasia, 21 in BE (Additional file 1: Figure S1).

#### Pathway analysis of differentially expressed mRNAs

Ingenuity Pathway Analysis (IPA) revealed an upregulation of the top 10 most significantly dysregulated canonical pathways in EAC with a down regulation of 9 out of 10 pathways in dysplasia (PPARa/RXRa upregulated), down regulation of the top 10 pathways in BE and down regulation of 9 out of 10 pathways in TANE (upregulation of PPAR) (Additional file 2: Figure S2).

#### Microbial profiling: Levrat model samples

**Fig. 3:**
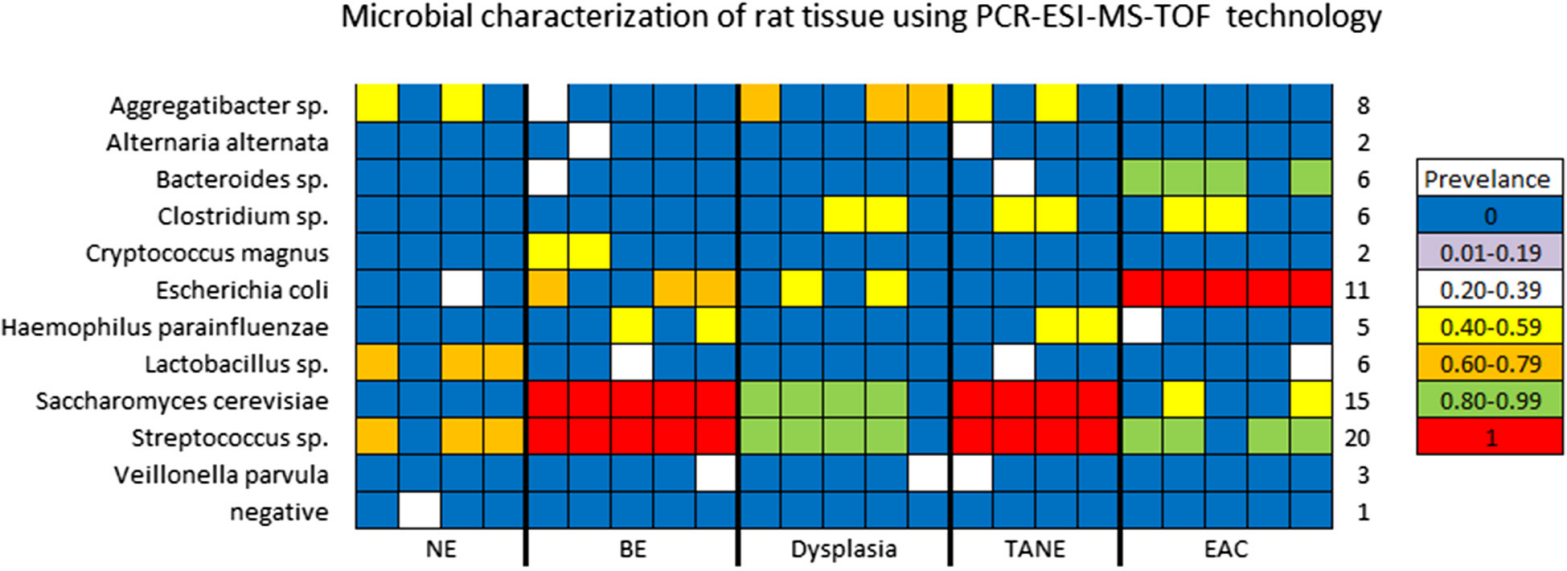
Microbial characterization of rat esophageal tissue using PCR-ESI-MS-TOF technology Bacteria were detected in 2 or more specimens in the Levrat animal model. Bacteria detected in only one specimen were not included. Each specimen is represented by one column. Rows represent bacterial species. Cells are color coded for prevalence of the bacteria within the experimental group

A diverse range of bacterial and fungal organisms were detected in both the Levrat esophageal specimens and the human clinical samples using the PCR-ESI-MS-TOF technology. Microbial profiling of the Levrat rat model detected microorganisms in all four experimental groups. Of the 23 samples tested, 22 Levrat esophageal specimens were positive for microbial DNA. Forty three different species of bacteria were detected in the animal model samples. The most prevalent microorganisms detected were *Aggregatibacter species* (8 out of 23)*, Escherichia coli* (11 out of 23), *Haemophilus parainfluenzae* (6 out of 23), *Saccharomyces cerevisiae* (15out of 23), *and Streptococcus species* (20 out of 23). While *Streptococcus species* where evenly distributed between the experimental groups, some bacteria were more prevalent within certain groups (Fig. 3). *Escherichia coli* was detected in 100 % of the EAC specimens (5 out of 5) in comparison to 25 % in the NE (1 out of 4), 60 % in the BE group (3 out of 5) and 40 % in the dysplasia group (2 out of 5). Also of interest *Clostridium species* was detected in 40 % of the TANE, dysplasia, and EAC groups but was absent from the NE and BE specimens. Additionally *Saccharomyces cerevisiae* was detected at a higher rate (80–100 %) within the dysplasia, TANE and BE specimens than in the NE and EAC (0–20 %).

#### Microbial profiling: human clinical samples

**Fig. 4:**
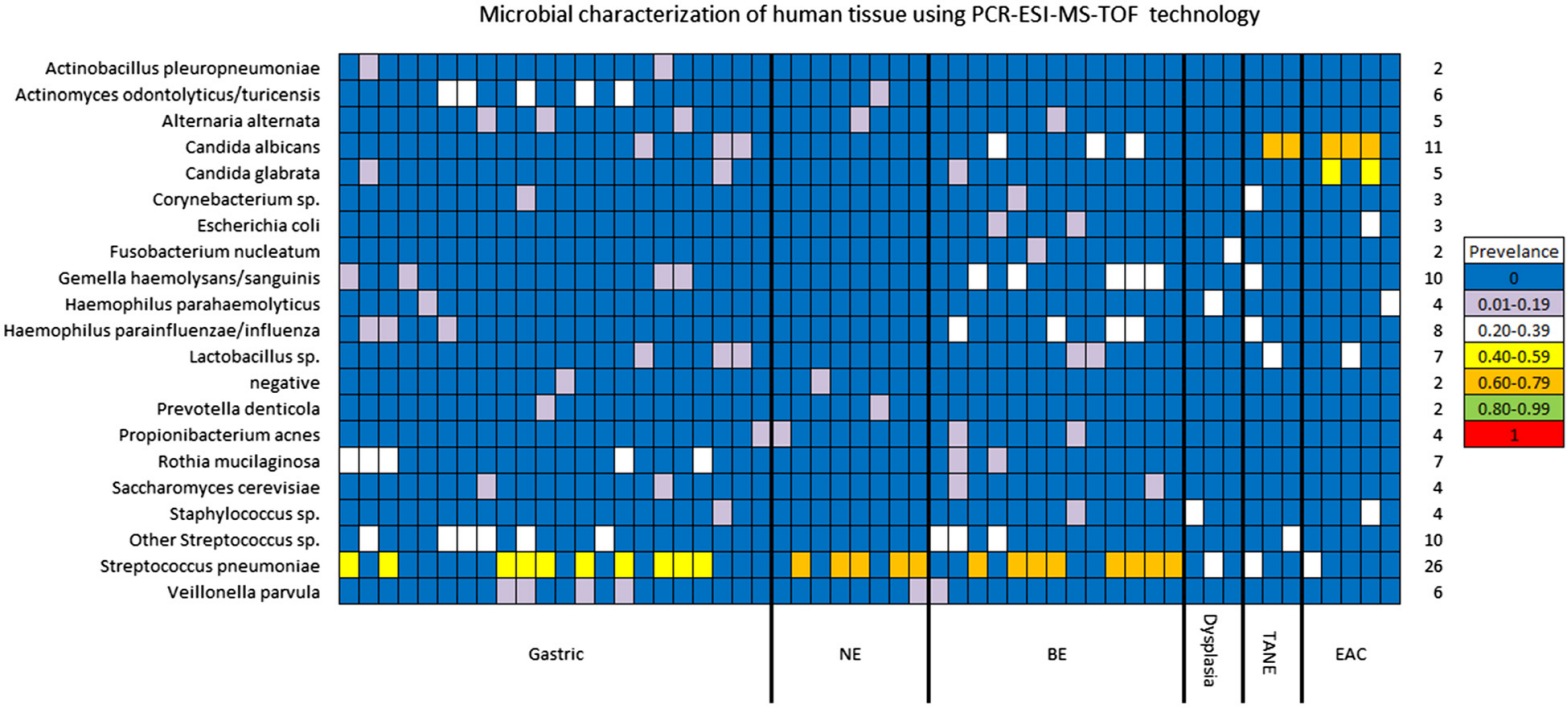
Microbial characterization of human esophageal tissue using PCR-ESI-MS-TOF technology. Bacteria were detected in 2 or more specimens in the human clinical samples. Bacteria detected in only one specimen were not included. Each specimen is represented by one column. Rows represent bacterial species. Cells are color coded for prevalence of the bacteria within the experimental group

**Fig. 5:**
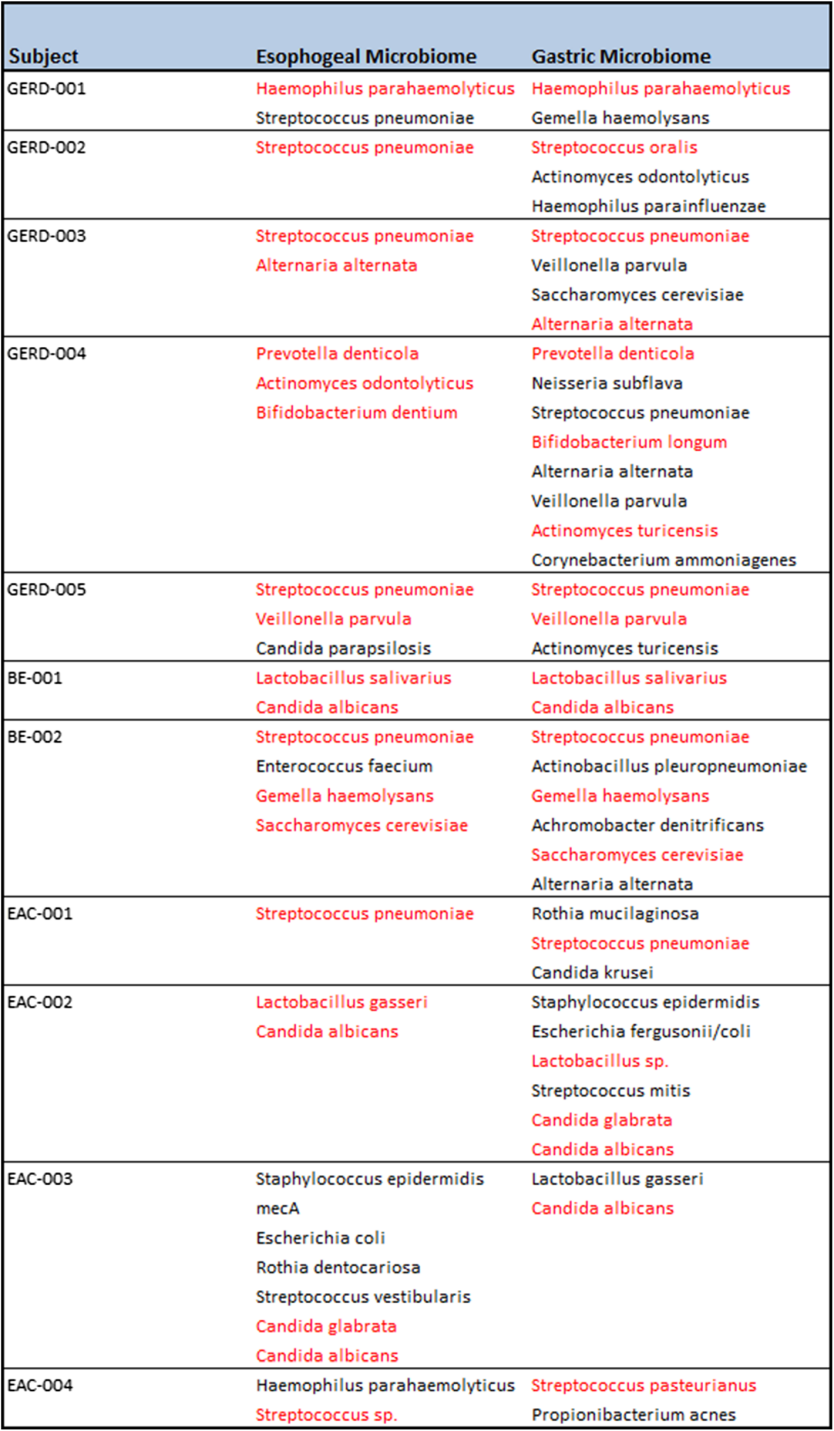
Comparison of Subject’s samples by location. Microrganisms highlighted in red are genus level match between both the esophageal and the gastric samples.

Microbial profiling of the 54 human clinical samples revealed microorganisms in all five experimental groups. Of the 54 samples tested, 52were positive for microbial DNA. The most prevalent microorganisms detected were *Candida sp.* (16 out of 54)*, Gemella sp.* (10 out of 54), *Haemophilus sp.* (12 out of 54), *and Streptococcus sp.* (36 out of 54). *Streptococcus pneumonia* was detected in high abundance in theGastric NE and BE groups (50–70 %) in comparison to TANE, dysplasia, and EAC (20–30 %). *Escherichia coli* was detected in the BE and EAC groups but was absent in the TANE, dysplasia, and the NE groups. *Actinomyces sp.* and *Veillonella parvula* were found almost exclusively in the Gastric specimens, while Gemella sp. was found predominantly in the BE specimens. Additionally *Candida albicans* and *Candida glabrata* was detected in more than half of the EAC samples (Fig. 4). Where tissue was available, we compared the microbiome of a subjects’ esophageal and gastric tissue. No correlation was seen within sample location however correlations within a subject’s specimens were observed. All 11 subjects with both esophageal and gastric tissue microbiome results had at least one genus level correlation between the two locations. Seven subjects had correlation values of 50 % or greater (Fig. 5).

#### Fish

**Fig. 6:**
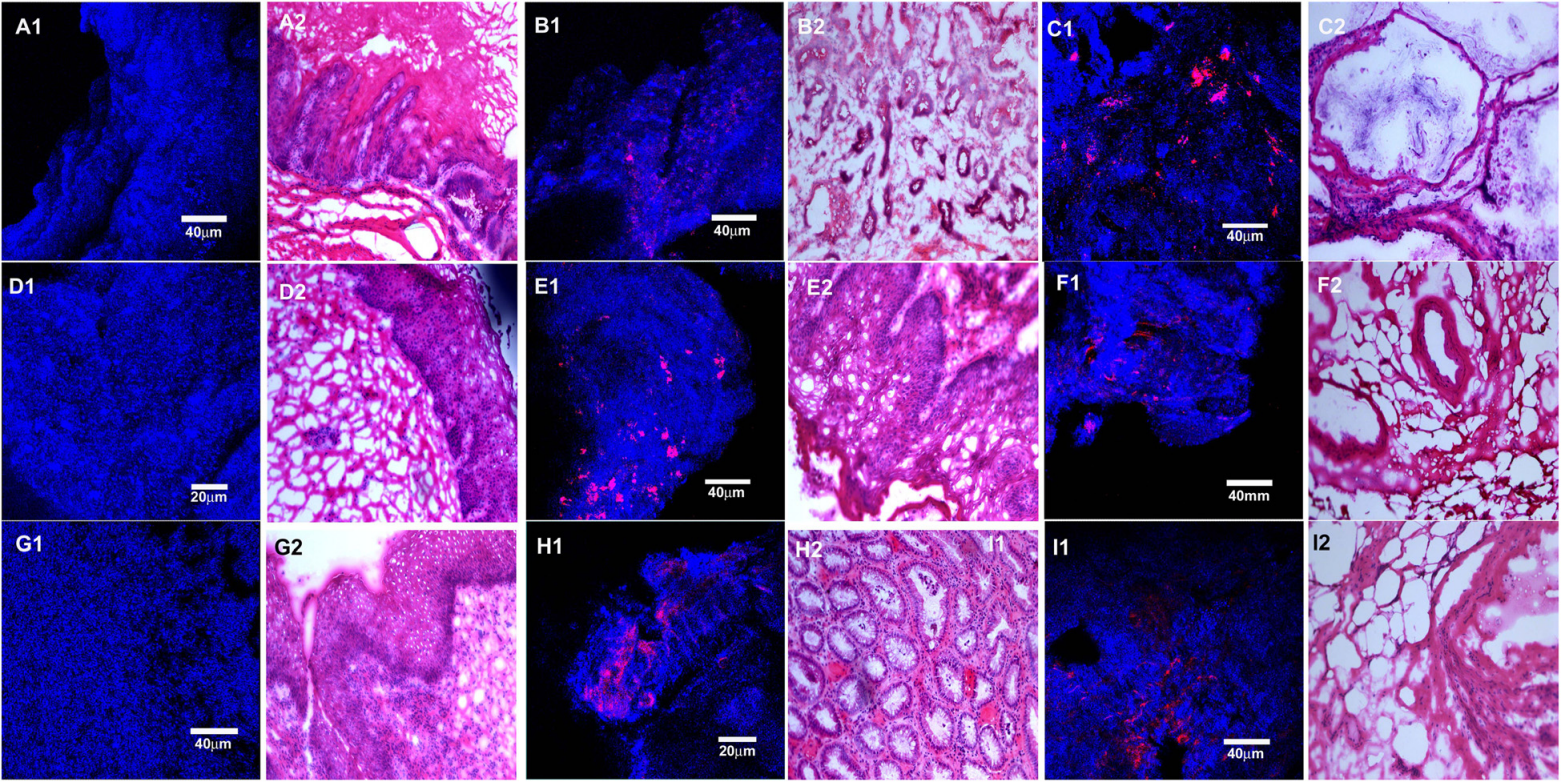
Biofilm bacteria adherent to rat and human tissues visualized by fluorescence in situ hybridization (FISH) probes (red). Blue is the reflected light from the tissue. A1-I1 represent FISH and A2-I2 represent the corresponding H&Es. A1,A2) Rat TANE tissue (negative control) shows absence of E.coli staining within the tissue B1, B2) Rat BE tissue shows presence of E.coli single cells and in clusters attached to the tissue; C1, C2) Rat EAC tissue shows presence of *E. coli* single cells and in clusters attached to the tissue; D1,D2) Human TANE tissue (negative control) shows absence of *S. pneumonia* staining within the tissue;E1,E2) Human BE tissue shows presence of *S. pneumonia* single cells and in clusters attached to the tissue; F1,F2) Human EAC tissue shows presence of *S. pneumonia* single cells and in clusters attached to the tissue; G1,G2) Human TANE tissue (negative control) shows absence of E.coli staining within the tissue; H1,H2) Human BE tissue shows presence of E.coli single cells attached to the tissue; I1,I2) Human EAC tissue shows presence of E.coli single cells and in clusters attached to the tissue. All H&Es are represented at 10X magnification

To confirm the PCR-ESI-MS-TOF results, where sufficient tissue was available, a secondary method of analysis was applied. One Levrat rat sample and four human clinical samples were stained by FISH and visualized by confocal microscopy (Fig. 6). The FISH probes were selected based on the species detected by the PCR-ESI-MS-TOF analysis, specifically *Escherichia coli* and *Streptococcus pneumonia.* This probe binds 16 s rRNA. Thus, the stained bacteria were metabolically active at the time of fixation and washing. This establishes that PCR-ESI-MS-TOF is detecting viable bacteria within the tissue at the time of collection.

## Discussion

Gene expression profiling revealed differential expression of TLRs in the 4 rat tissues of interest compared to the normal rat epithelium (NE): EAC, dysplasia, BE and TANE. TLRs 1-3, 6, 7 and 9 are significantly upregulated in EAC while TLR 1 was significantly upregulated in TANE, BE and dysplasia. AThis was further corroborated by incremental dysregulation of a number of genes as noted in the TLR signaling pathway (21, 27 and 31 for BE, dysplasia and EAC respectively). Activation of TLRs along with dysregulation of target genes in this pathway were confirmed by quantitative RT-PCR and the differential presence of *E. coli* and *S. cerevisiae* across the EAC spectrum was confirmed by PCR-ESI-MS-TOF and validated by FISH. These results suggest an active role for microbial signaling in EAC carcinogenesis using the Levrat’s model.

TLRs are pattern recognition receptors integral to innate immunity. Their link to tumorigenesis is increasingly being recognized [13]. Expression of TLRs is noted in various effectors of the immune system as well as tissues exposed to the external environment, e.g. the gastrointestinal tract. They can be both extracellular (e.g. TLR4) to recognize molecules that they may be exposed to or intracellular (e.g. TLR9) to detect nucleic acids that would be important in host responses to viruses and certain bacteria [14–16]. Binding of ligands to TLRs and signaling can potentially lead to activation of two pathways involving different adaptor molecules: MyD88-dependent and MyD88-independent. The MyD88-dependent pathway leads to production of inflammatory cytokines, whereas the MyD88-independent pathway is associated with the stimulation of IFN-β and the maturation of dendritic cells [25]. To date there are 13 different TLRs recognized and differential expression of these molecules has been noted on normal esophageal epithelium [26] as well as EAC [27]. Being exposed to pathogens from the external environment on a daily basis, there is rationale to explore the potential role of these pathogens in innate immunity through TLR mediation in EAC [28].

We used the modified Levrat surgical model to study EAC. The model uses an end-to-side esophagojejunal anastomosis and is highly efficient for inducing tumorigenesis with an observed 70 % rate of adenocarcinoma development at 28 weeks after surgery. Previous studies using this rat model have shown that the resultant gastroduodenojejunal reflux leads to a reliable progression from BE to EAC on a histologic and molecular level [29]. This reliable progression from BE to EAC allowed us to study and identify pathways differentially dysregulated in the metaplasia-dysplasia-carcinoma sequence for EAC. As noted in Fig. 4, we identified upregulation in the top 10 most significantly dysregulated canonical pathways in EAC with a down regulation of 9 out of 10 pathways in dysplasia (PPARa/RXRa upregulated), down regulation of the top 10 pathways in BE, and down regulation of 9 out of 10 pathways in TANE (upregulation of PPAR).

Interestingly, prevalence of *E. coli* based on PCR-MS-ESI-TOF analysis was noted in 60 % of cases of BE and 100 % of EAC in the Levrat model, which was validated by FISH. *E.coli* infection in the urinary tract has been shown to cause chronic inflammation, produce histological changes within host tissue, and promote bladder lesions within rats. *E.coli* infection alone produced elevated levels of IL-6 within bladder tissue similar to the levels demonstrated in urinary tract inflammatory diseases and cancer [30]. Elevated IL-6 was also detected in our Levrat model across the EAC spectrum. Three mechanisms have been suggested for the role of *E .coli* in tumor promotion: (a) the bacteria increase the carcinogenic potential of nitrosamine precursors, (b) accelerate urothelial proliferation, and (c) expose the host tissue to free radicals which results in DNA damage. *E .coli* derived outer membrane vesicles have been shown to be genotoxic regardless of the strain’s pathogenicity [31].

In the human clinical samples, esophageal tissue is continuously exposed to swallowed commensal bacteria from the oral cavity. Bacteria located within saliva include: *Streptococcus, Neisseria, Veillonella, Fusobacterium, Bacteriodes, Lactobacillius*, *Staphylococcus*, and *Enterobacteriaceae* [32]. While normally harmless, several species of *Streptococcus* are being investigated in relationship to the development of esophageal cancer. Streptococcus is an invasive bacterium which targets host fibronectin and induces a cytokine response. These actions have the potential to promote inflammation, cause dysphagia, and promote carcinogenesis [33]. Our high-level detection of *Streptococcus pneumonia* in the NEand BE groups (50–70 %) in comparison to TANE, dysplasia, and EAC (20–30 %) hints towards these observations. *Streptococcus* may be causing inflammation and damage in the NE and BE groups but once carcinogenesis has occurred the bacteria leaves the damaged tissue to invade surrounding healthy tissue.

While several studies have screened esophageal diseases for microorganisms through the use of culture and targeted molecular technologies, the application of a broad technology, PCR-ESI-MS-TOF, allows researchers to screen specimens without bias. Studying microbiomes and their potential role in diseases like GERD and EAC opens up a new approach to studying their pathogenesis and identifying new therapeutic targets [34].

Major limitations of our study include the limited sample size and focused analysis on the selected group of microbiota as noted. It would be important to study in detail other representative groups of microbiomes in EAC models. Additionally, the pilot study established the need for a follow on prospective human trial to delineate the true role of microbiota and TLRs, in both disease development and progression. As the small sample size and the cross-sectional design of the current study are limitations for making strong inferences about the role of individual microbiomes and TLR associations, for a heterogenetic cancer. Lastly, our work lays the foundation for studying the in depth the molecular mechanisms in play between specific microbiata and associated TLRs. .

## Conclusions

The associations demonstrated between the TLR signaling pathway and *E. coli* and *S. cerevisiae* hint towards possible early molecular changes being mediated through the microbiome in a rat-model of BE-EAC sequence. Human clinical samples did corroborate the results to some extent; however, studies with larger sample size would be needed to better explore this association.

## Additional files

Additional file 1:
**Figure S1.** Venn diagram showing the number of significantly dysregulated genes in the TLR signaling pathway across the EAC spectrum. (TIF 511 kb) Additional file 2:
**Figure S2.** Ingenuity Pathway Analysis (IPA) of the top ten most significantly dysregulated canonical pathways across the EAC spectrum. (TIF 659 kb)

## Abbreviations

BE: barrett’s esophagus
DAMPs: danger-associated molecular patterns
*E. coli*: *Escherichia coli*
EAC: esophageal adenocarcinoma
ESI: electrospray ionization
FISH: fluorescent in situ hybridization
IECs: intestinal epithelial cells
IPA: ingenuity pathway analysis
MS: mass spectrometry
NE: normal epithelium
PAMPs: pathogen-associated microbial patterns
PCR: polymerase chain reaction
*S. pneumonia*: *Streptococcus pneumoniae*
*Sp.*: *Species*
TANE: tumor adjacent normal epithelium
TLR: toll-like receptor
TOF: time of flight
