# Supplementary figures and images for: Associations of microbiota and toll-like receptor signaling pathway in esophageal adenocarcinoma
Source: BMC Cancer. 2016 Feb 2;16:52. doi: 10.1186/s12885-016-2093-8 (PMC4739094; doi:10.1186/s12885-016-2093-8)

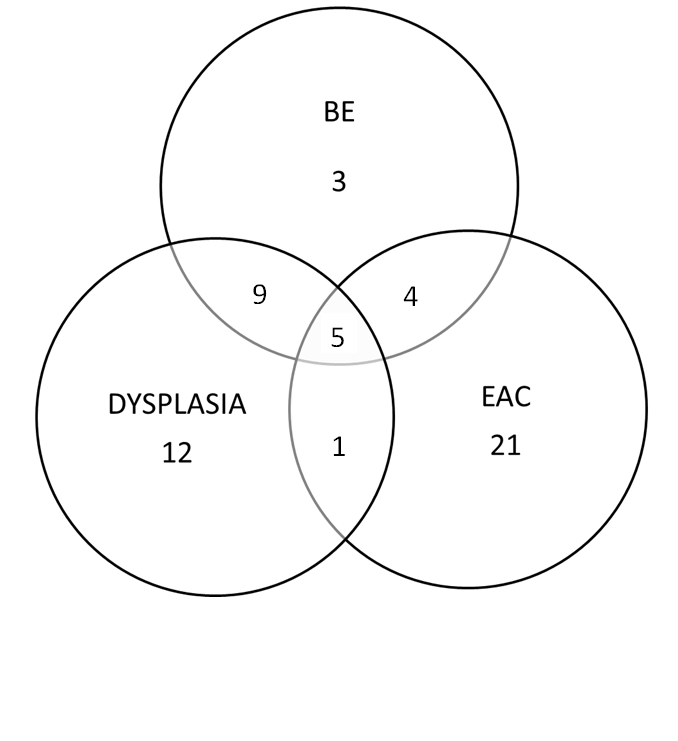

Supplement: Additional file 1: — Figure S1. Venn diagram showing the number of significantly dysregulated genes in the TLR signaling pathway across the EAC spectrum. (TIF 511 kb) [file 12885_2016_2093_MOESM1_ESM.tif]

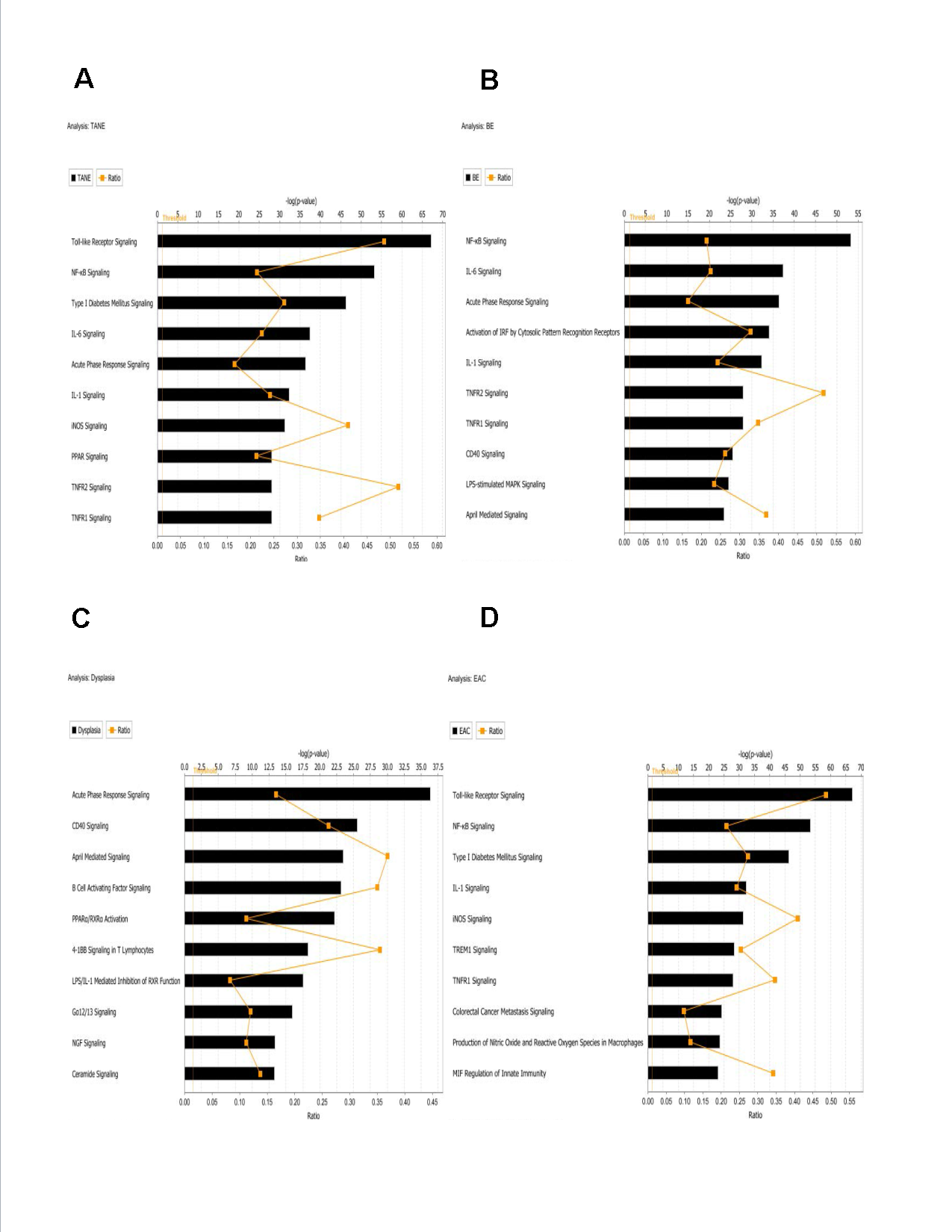

Supplement: Additional file 2: — Figure S2. Ingenuity Pathway Analysis (IPA) of the top ten most significantly dysregulated canonical pathways across the EAC spectrum. (TIF 659 kb) [file 12885_2016_2093_MOESM2_ESM.tif]
